# Supplementary material for: Formation of ammonia–helium compounds at high pressure
Source: Nat Commun. 2020 Jun 22;11:3164. doi: 10.1038/s41467-020-16835-z (PMC7308345; doi:10.1038/s41467-020-16835-z)
Supplement: Supplementary file 1 — Supplementary Information [file 41467_2020_16835_MOESM1_ESM.pdf]

Supplementary Information

Formation of ammonia–helium compounds at high pressure

Shi *e*t al.

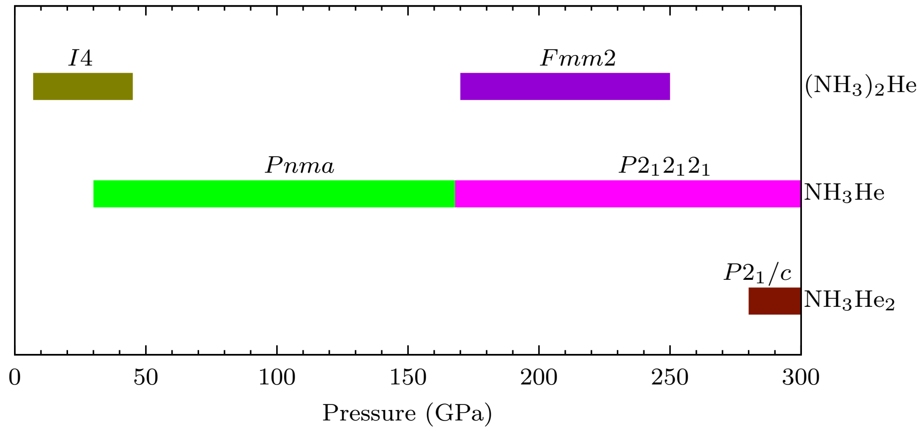

Supplementary Figure 1: Phases stability ranges for binary ammonia–helium mixtures as a function of pressure. For addition, we did not consider the zero point vibration energy for this calculation. Stable compounds are labeled by space groups.

Supplementary Table 1: Predicted crystal structures of the $I4$ phase of $(NH_3)_2He$ at 10 GPa. the $Fmm2$ phase of $(NH_3)_2He$ at 180 GPa, the $Pnma$ phase of NH_3He at 35 GPa, the $P2_12_12_1$ phase of NH_3He at 180 GP and the $P2_1/c$ Phase of NH_3He_2 at 240 GPa.

Pressure (GPa)	Space group Compound	Lattice parameters	Atomic coordinates (fractional)
10	$I4$	$a = b$ =6.04790 Å	N (8c) (0.68699 0.81532 0.49000)
	$(NH_3)_2He$	c=5.4586 Å	H (8c) (0.71540 0.21482 0.80645)
		α=β=γ=90°	H (8c) (0.51931 0.21518 0.01598)
			H (8c) (0.71058 0.02184 0.01902)
			He (2a) (0.00000 0.00000 0.25678)
			He (2a) (0.00000 0.00000 0.76731)
180	$Fmm2$	a=4.12910 Å	N (4a) (-0.00000 0.00000 -0.62411)
	$(NH_3)_2He$	b=6.54400 Å	N (4a) (0.00000 0.00000 -0.96256)
		c=6.77250 Å	N (8c) (0.50000 0.17218 -0.79512)
		α=β=γ=90°	H (16e) (0.19086 0.60707 -0.73383)
			H (8c) (-0.00000 0.85860 -0.69604)
			H (8c) (0.00000 0.13405 -0.88820)
			H (8c) (0.00000 0.38813 -0.93367)
			H (8d) (0.78664 -0.00000 -0.04216)
			He (8b) (0.25000 0.25000 -0.54269)
35	$Pnma$	a=5.62190 Å	N (4c) (-0.18995 0.75000 -0.15194)
	NH_3He	b=3.55170 Å	H (8d) (0.28444 0.97511 -0.49195)
		c=4.33250 Å	H (4c) (-0.01325 0.75000 -0.20685)
		α=β=γ=90°	He (16e) (0.19086 0.60707 -0.73383)
180	$P2_12_12_1$	a=3.9222 Å	N (4a) (0.86438 0.99549 0.07317)
	NH_3He	b=2.6638 Å	H (4a) (0.90937 0.31647 0.46663)
		c=4.8822 Å	H (4a) (0.60088 0.64555 0.54120)
		α=β=γ=90°	H (4a) (1.19702 0.46259 0.23006)
			He (4a) (1.10390 0.01888 0.73228)
240	$P2_1/c$	a=4.83640 Å	N (4c) (-0.26039 0.86854 0.49078)
	NH_3He_2	b=3.68990 Å	H (4e) (-0.26635 0.78100 0.69548)
		c=4.66630 Å	H (4e) (-0.52641 0.54643 0.18694)
		α=γ=90°	H (4e) (-0.03196 0.02352 0.65124)
		β=134.17°	He (4e) (-0.15373 0.13108 -0.07396)
			He (4e) (-0.66663 0.62963 -0.58984)

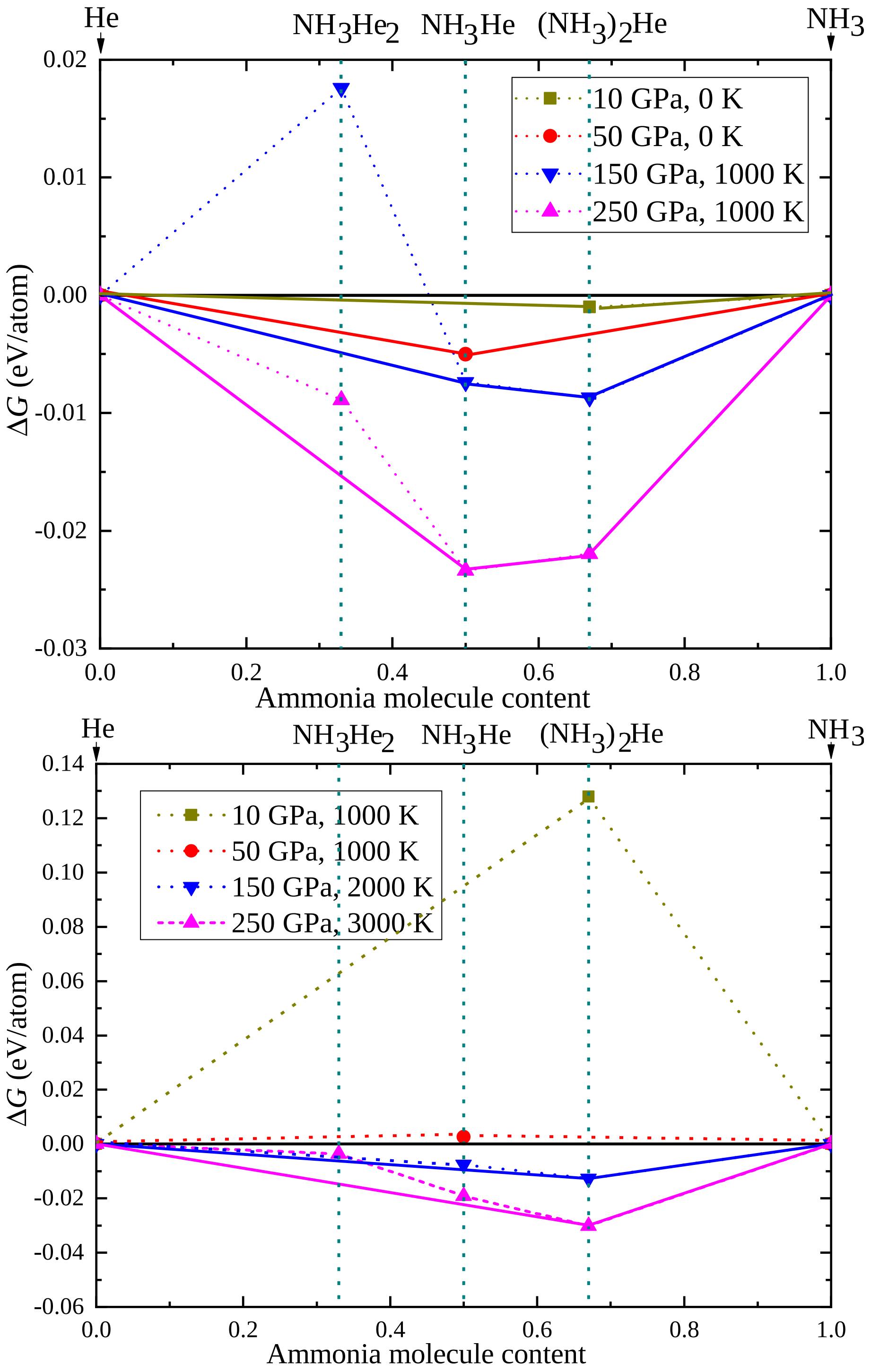

Supplementary Figure 2: Calculated Gibbs free energies (ΔG in eV/atom) of various NH_3–He compounds with respect to the decompositions at 10 GPa (0 K and 1000 K), 50 GPa (0 K and 1000 K), 150 GPa (1000 K and 2000 K) and 250 GPa (1000 K and 3000 K), respectively. The solid lines represent the convex hulls, while the dotted lines are just a guide for the eyes.

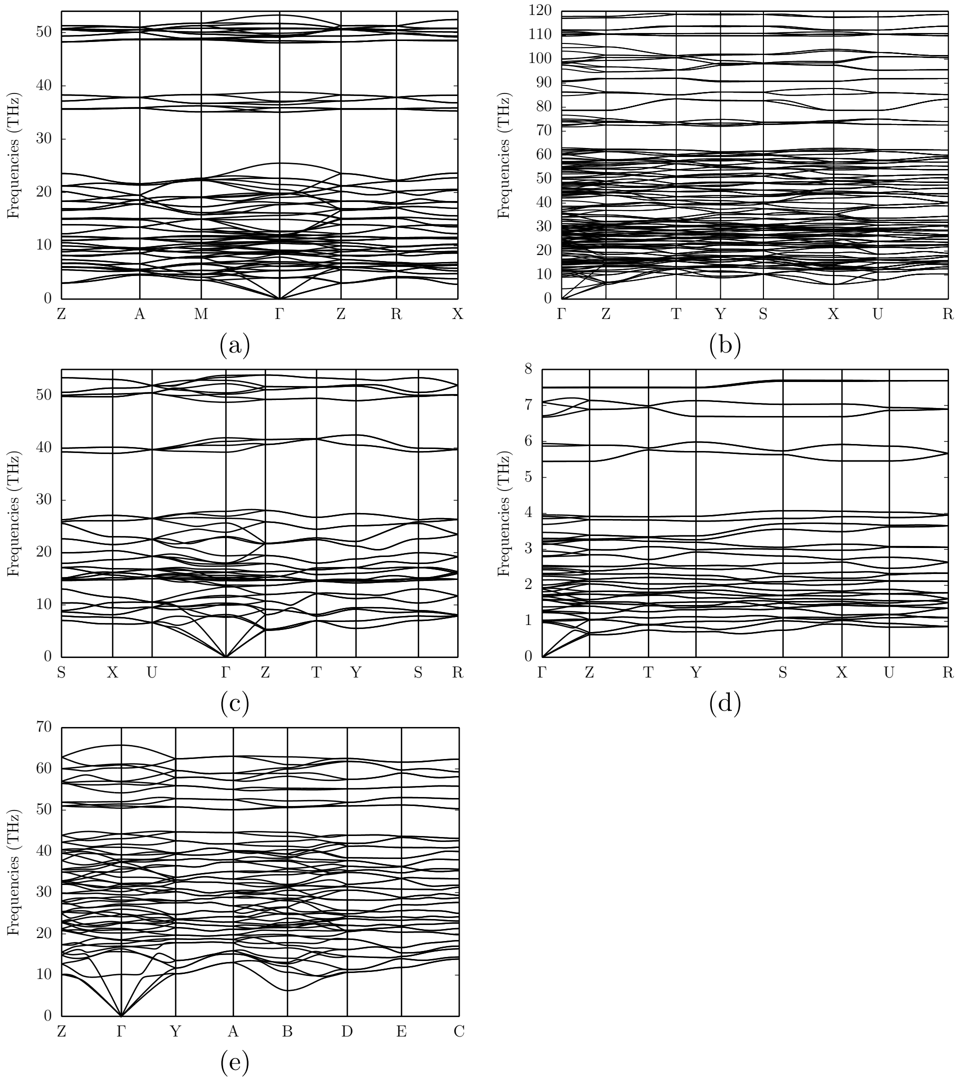

Supplementary Figure 3: The phonon dispersions for (a) the $I4$ phase of $(NH_3)_2He$ at the 10 GPa, (b) the $Fmm2$ phase of $(NH_3)_2He$ at the 180 GPa, (c) the $Pnma$ phase of NH_3He at the 35 GPa, (d) the $P2_12_12_1$ phase of NH_3He at the 180 GPa and (e) the $P2_1/c$ Phase of NH_3He_2 at the 300 GPa.

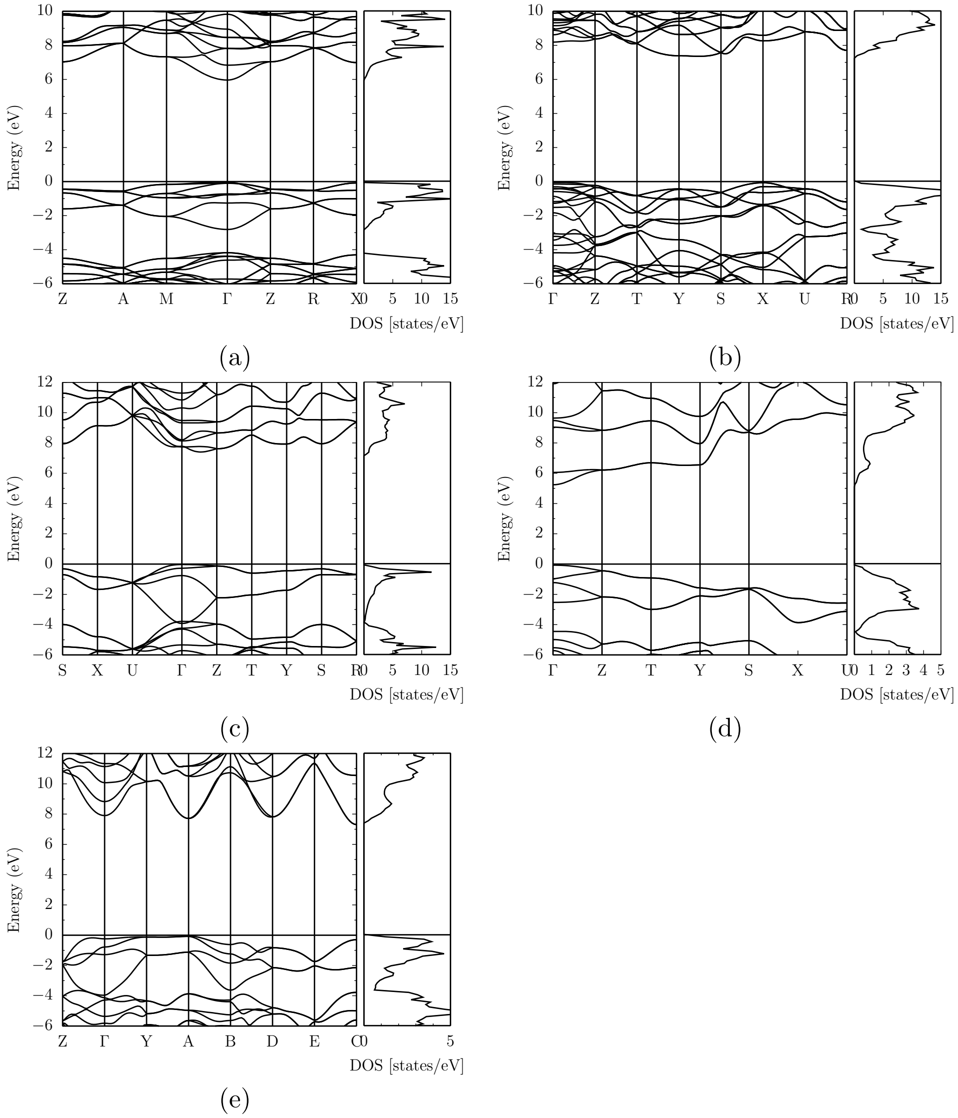

Supplementary Figure 4: The band structures and density of states: (a) the $I4$ phase of $(NH_3)_2He$ at 10 GPa, (b) the $Fmm2$ phase of $(NH_3)_2He$ at 180 GPa, (c) the $Pnma$ phase of NH_3He at 35 GPa, (d) the $P2_12_12_1$ phase of NH_3He at 180 GPa, (e) the $P2_1/c$ Phase of NH_3He_2 at 300 GPa.

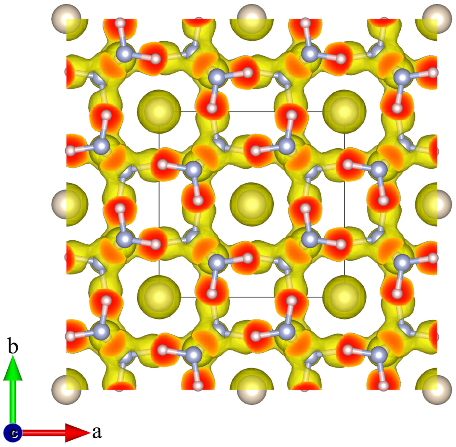

(a) I_4 phase of $(NH_3)_2He$

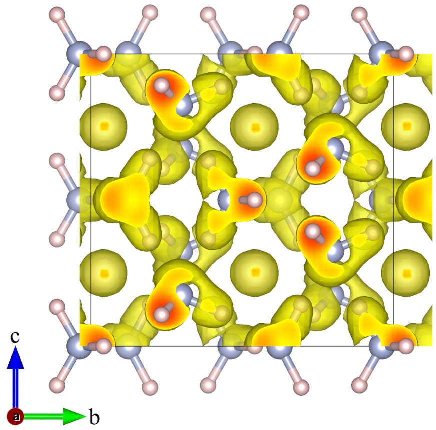

(b) $Fmm2$ phase of $(NH_3)_2He$

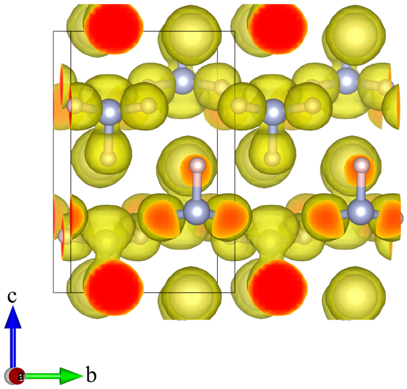

(c) $Pnma$ phase of NH_3He

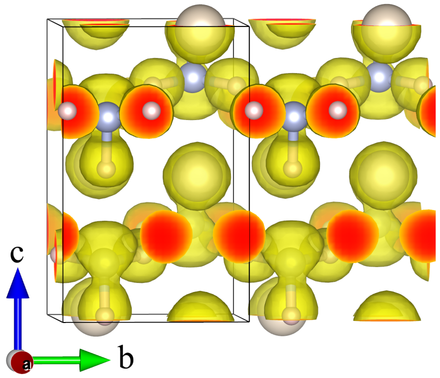

(d) $P2_12_12_1$ phase of NH_3He

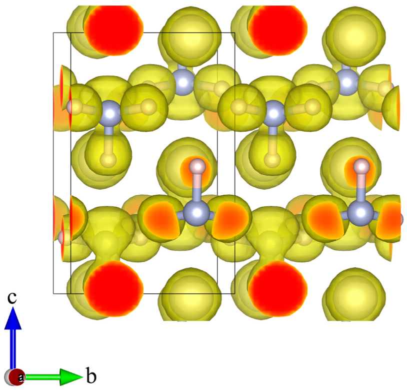

(e) $P2_1/c$ phase of NH_3He_2

Supplementary Figure 5: Three-dimensional electron localization function with isosurface value of 0.8 of (a) The $I4$ phase of $(NH_3)_2He$ at 10 GPa, (b) The $Fmm2$ phase of $(NH_3)_2He$ at 180 GPa, (c) The $Pnma$ phase of NH_3He at 35 GPa, (d) The $P2_12_12_1$ Phase of NH_3He at 180 GPa and (e) The $P2_1/c$ Phase of NH_3He_2 at 300 GPa, respectively.

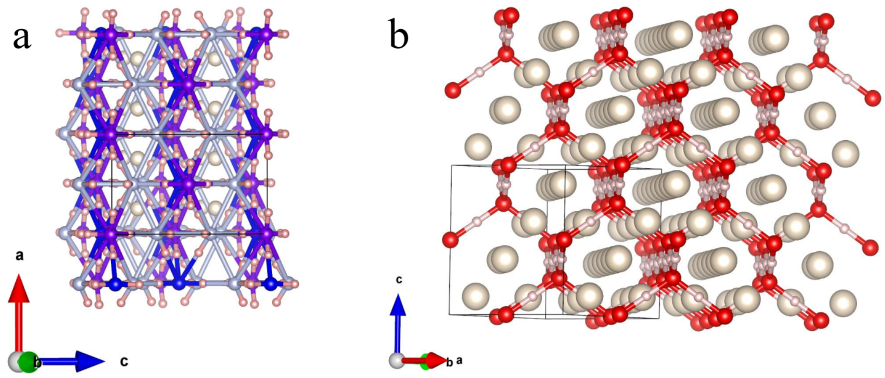

Supplementary Figure 6: The crystall structures of (a) $Fmm2$ phase of $(NH_3)_2He$ and (b) $Fm\bar{3}m$ phase of He_2H_2O

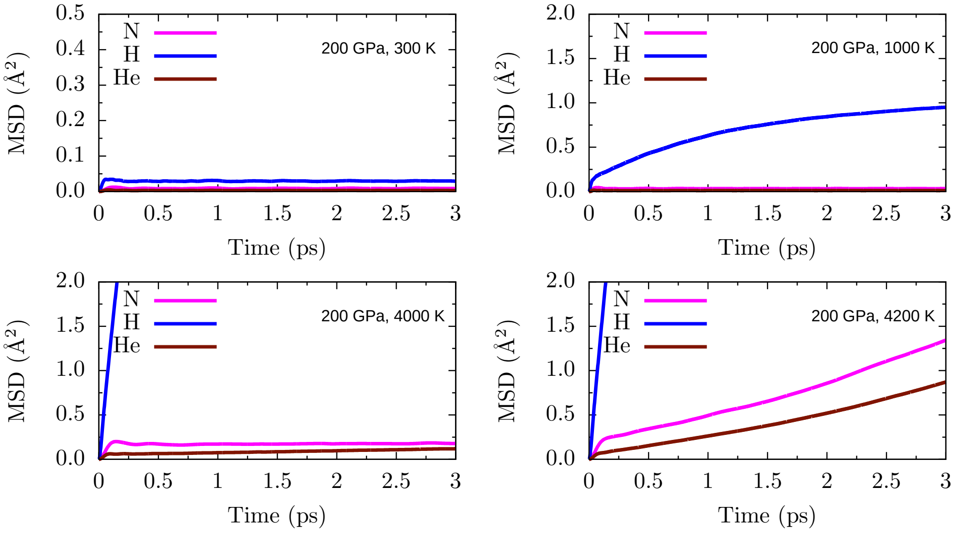

Supplementary Figure 7: The calculated mean squared displacement (MSD) of the atomic positions of $Fmm2$-$(NH_3)_2He$ phase at pressure of 200 GPa and temperature of 300 K, 1000 K, 4000 K and 4200 K, respectively.

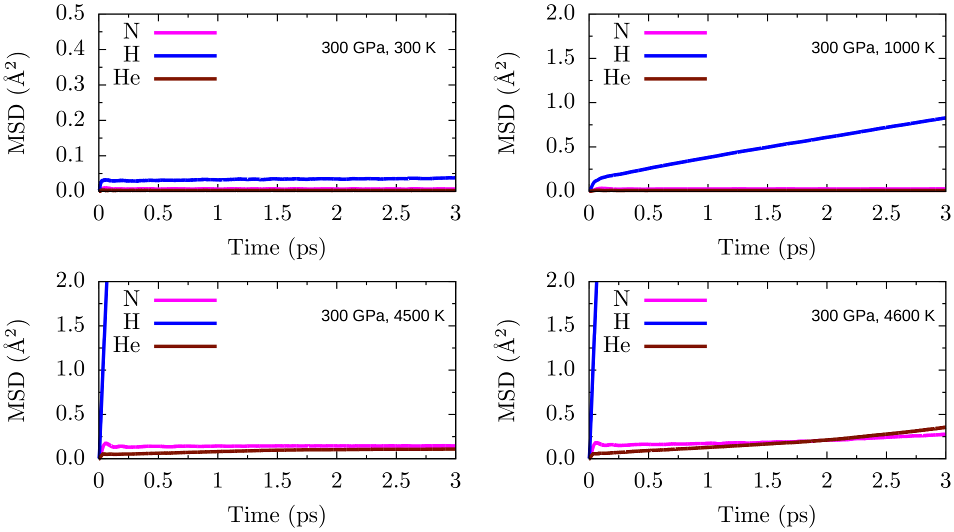

Supplementary Figure 8: The calculated mean squared displacement (MSD) of the atomic positions of $Fmm2$-$(NH_3)_2He$ phase at pressure of 300 GPa and temperature of 300 K, 1000 K, 4500 K and 4600 K, respectively
